# Biopolymer Blends of Poly(lactic acid) and Poly(hydroxybutyrate) and Their Functionalization with Glycerol Triacetate and Chitin Nanocrystals for Food Packaging Applications

**DOI:** 10.1021/acsapm.2c00967

**Published:** 2022-08-16

**Authors:** Mitul Kumar Patel, Freja Hansson, Olli Pitkänen, Shiyu Geng, Kristiina Oksman

**Affiliations:** † Division of Materials Science, Department of Engineering Sciences and Mathematics, Luleå University of Technology, SE-97 187 Luleå, Sweden; ‡ Microelectronics Research Unit, Faculty of Information Technology and Electrical Engineering, University of Oulu, 90570 Oulu, Finland; § Mechanical & Industrial Engineering (MIE), University of Toronto, Toronto, Ontario M5S 3G8, Canada; || Wallenberg Wood Science Center (WWSC); Luleå University of Technology, SE 97187 Luleå, Sweden

**Keywords:** poly(lactic acid), poly(hydroxybutyrate), chitin nanocrystals, nanocomposites, crystallization, morphology, microscopy, barrier properties

## Abstract

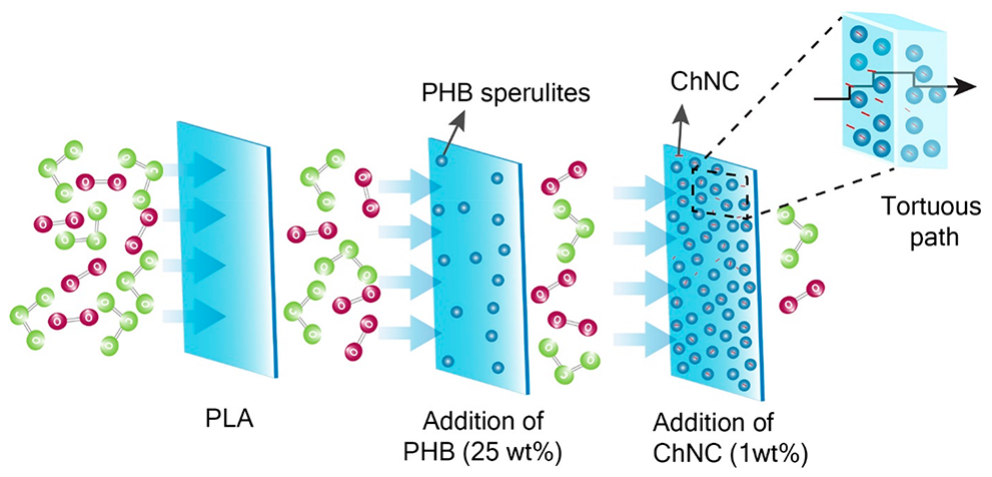

Polylactic acid (PLA) is a biopolymer that has potential for use in food packaging applications; however, its low crystallinity and poor gas barrier properties limit its use. This study aimed to increase the understanding of the structure property relation of biopolymer blends and their nanocomposites. The crystallinity of the final materials and their effect on barrier properties was studied. Two strategies were performed: first, different concentrations of poly(hydroxybutyrate) (PHB; 10, 25, and 50 wt %) were compounded with PLA to facilitate the PHB spherulite development, and then, for further increase of the overall crystallinity, glycerol triacetate (GTA) functionalized chitin nanocrystals (ChNCs) were added. The PLA:PHB blend with 25 wt % PHB showed the formation of many very small PHB spherulites with the highest PHB crystallinity among the examined compositions and was selected as the matrix for the ChNC nanocomposites. Then, ChNCs with different concentrations (0.5, 1, and 2 wt %) were added to the 75:25 PLA:PHB blend using the liquid-assisted extrusion process in the presence of GTA. The addition of the ChNCs resulted in an improvement in the crystallization rate and degree of PHB crystallinity as well as mechanical properties. The nanocomposite with the highest crystallinity resulted in greatly decreased oxygen (O*)* and carbon dioxide (CO_2_) permeability and increased the overall mechanical properties compared to the blend with GTA. This study shows that the addition ChNCs in PLA:PHB can be a possible way to reach suitable gas barrier properties for food packaging films.

## Introduction

Poly(lactic acid) (PLA) is a popular biobased polymer for packaging applications owing to its superior mechanical properties,^1^ processability,^2^ transparency, excellent printability,^3^ and economic feasibility compared to other biodegradable polymers. PLA is currently utilized in various packaging applications such as plates, cups, lids, and drinking straws, as well as bags and film packaging.^4^

Despite its many benefits, PLA has significant limitations that restrict its usage in food packaging, such as low ductility, low crystallinity, and moderate barrier performance.^5^ In particular, the barrier performance of a material is an important factor for maintaining the shelf life of the packaged products.^6^ The degree of crystallinity and molecular characteristics of PLA considerably influence its barrier performance.^7^ Several strategies have been used to improve the degree of crystallinity, such as blending with other polymers^8−11^ or use of biobased nanomaterials as nucleation agents.^12−14^ Blending PLA with a high crystalline biobased and biodegradable polymer such as poly(hydroxybutyrate) (PHB) can improve the overall crystallinity and, hence, the barrier properties desirable in food packaging applications. PHB is a biobased and biodegradable polyester; it exhibits a high degree of crystallinity due to polymerization that results in the formation of macromolecules with a highly ordered stereochemical structure.^15−17^ However, owing to its high cost, brittleness, thermal instability, and poor melt viscosity, the use of pure PHB in food packaging is restricted.^9^ Recently, several studies have explored PLA:PHB blends for food packaging applications.^18−22^ To be miscible, two polymers must have similar solubility parameters (δ). The δ of PLA is 19.5–20.5 MPa^1/2^, while that of PHB is 18.5–20.1 MPa^1/2^ and good miscibility is expected due to the similar δ values of these two polymers.^19^ However, in addition to the solubility parameters, the miscibility of PLA and PHB also depends on the molecular weight^23−25^ and concentration of PLA.^26^ The processing temperature also plays an important role in the PLA–PHB performance.

The use of biobased nanomaterials such as chitin nanocrystals (ChNCs) have been shown to act as a nucleating agent for PLA, if these nanocrystals are well-dispersed in the matrix, and it is another technique for the enhancement of PLA crystallinity for packaging applications.^12^ The ChNCs are isolated from chitin, which is one of the most abundant biopolymers on earth and is the main structural component in exoskeletons of crustaceans such as shrimps, crabs, and lobsters. Several studies have been published on the laboratory-scale preparation of ChNCs from various raw materials using HCl acid hydrolysis.^27,28^ However, if the hydrolysis is done on a large scale, H_2_SO_4_ is a more suitable acid to hydrolyze the chitin because HCl is a strong reducing acid, which makes it highly corrosive when in contact with industrial equipment. It was shown in EU Horizon 2020 project Newpack that chitin nanocrystals can be produced with H_2_SO_4_;^29^ however, their colloidal stability is not as good due to the low negative surface charge.

In addition to the crystallinity improvement, additions of ChNCs have been shown to act as reinforcement and improve the mechanical and thermal properties of PLA.^14,30,31^ However, to the best of our knowledge, isolation of ChNCs at a larger scale using H_2_SO_4_ hydrolysis and its uses in PLA:PHB blends for the processing of nanocomposites with a scalable liquid-assisted extrusion approach and the effect of the ChNCs on the blend crystallization and barrier properties have not yet been reported.

The main objective of this study was to understand the structure–property relations of the fabricated PLA films that can lead to a high crystallization rate, high crystallinity, and low gas permeability. A two-step approach was assessed: first, the optimal composition of binary PLA:PHB blends with respect to crystallization, morphology, and thermal behavior was determined. Then, the optimal PLA:PHB blend was used for the addition of ChNCs as nucleation agents to obtain a further improvement in the crystallization. The effects of the ChNCs on the nanocomposites’ crystallization, thermal, mechanical, and gas barrier properties were studied.

## Experimental Section

### Materials

Extrusion grade PLA (Ingeo 4043 D, NatureWorks) with an average molecular weight (MW) of 200000 g mol^–1^ was purchased from Resinex Switzerland AG (Freienbach, Switzerland). Both PHB and ChNCs were produced on a pilot scale at the Bio Base Europe Pilot Plant (BBEPP, Belgium) as described in the previously reported study.^29^ Briefly, PHB was produced by the fed-batch fermentation of glucose syrup using *Parabulkholderia sacchari* at 29 °C, followed by downstream processing that involves cell lysis and enzymatic hydrolysis of the bacterial intracellularly stored granules and then concentration and grinding to form PHB powder. The purity of the produced PHB was higher than 95%. The ChNCs were isolated from shrimp chitin (Glentham Life Sciences, Ltd., Corsham, U.K.) via acid hydrolysis treatment. Shrimp chitin was diluted in 35 wt % H_2_SO_4_ and heated at 60 °C for 2 h in a Pfaudler AE 400 glass-lined reactor (Thaletec GmbH, Thale, Germany). The hydrolyzed chitin was then centrifuged, dialyzed, and homogenized to produce ChNCs. Atomic force microscopic (AFM) images (phase and height) of the used ChNCs are presented in Figure S1 in the Supporting Information. The AFM height image was processed in Gwyddion^32^ image analysis software (open source, version 2.52) to flatten with first order leveling and to remove skipping lines. The length and height for at least 100 ChNCs were measured. The width (height) and length ranges of the isolated ChNCs were measured between 5 and 16 nm (average 12 nm) and 120–480 nm (average, 286 nm), respectively. The supplied ChNCs at 4 wt % were concentrated to approximately 18 wt % using a vacuum rotary evaporator.

Glycerol triacetate (GTA) processing aid (≥99%; MW, 218 g/mol) was purchased from Sigma-Aldrich (Stockholm, Sweden), and the ethanol (99.5%) used in the suspension mixture was purchased from Solveco AB (Rosersberg, Sweden).

### Biopolymer Blends and Nanocomposite Preparation

**Figure 1:**
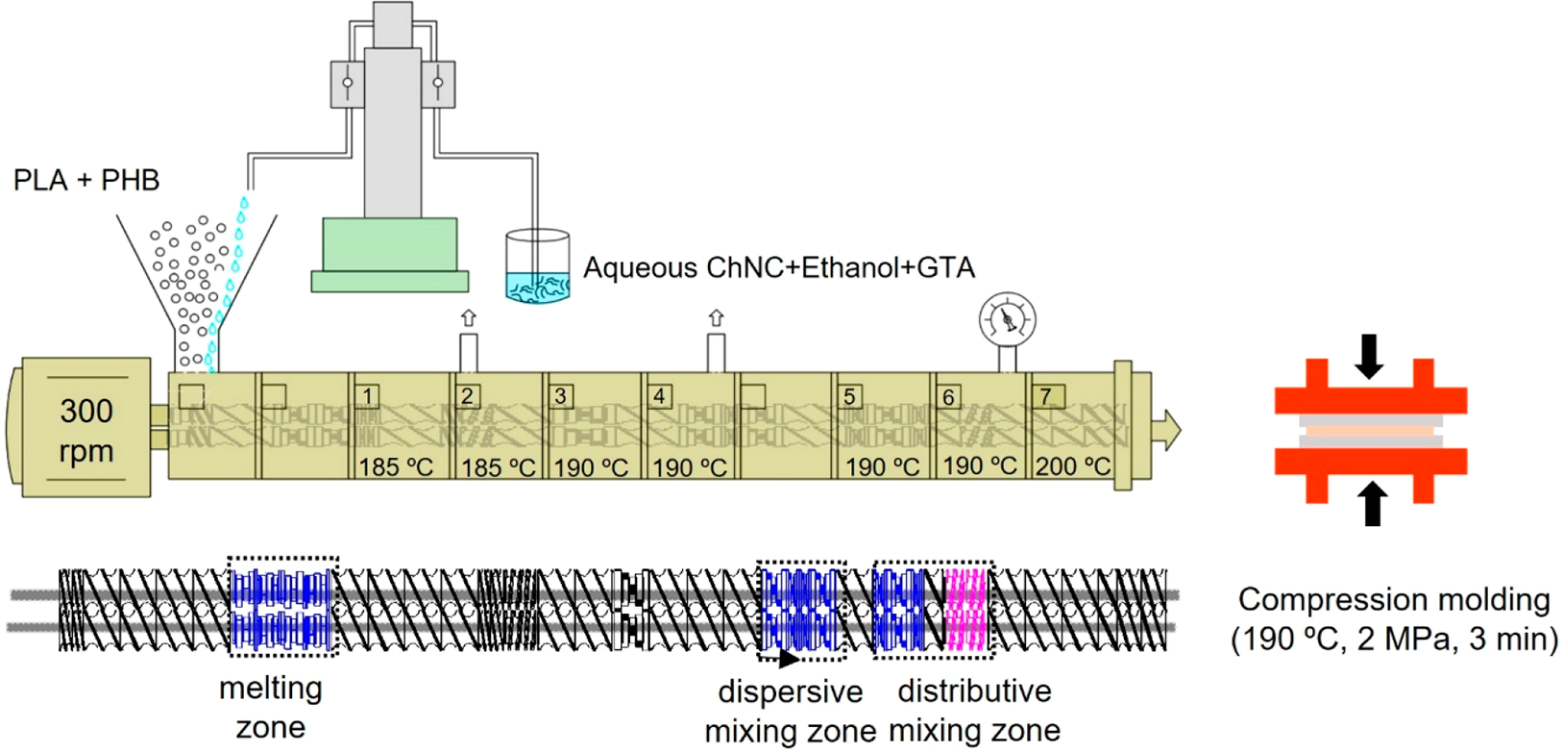
Schematic of the used twin-screw extrusion process, showing the modular design of the extruder with the screw configuration, used temperature profile, and pumping of the ChNC suspension and the compression molding of the pellets into films.

Both the blends and the nanocomposites were prepared in a co-rotating twin-screw extruder (Coperion W&P ZSK-18 MEGALab, Stuttgart, Germany) equipped with a Coperion K-Tron gravimetric feeder (Niederlenz, Switzerland). The polymers were dried overnight in a hot air oven at 55 °C. PLA and PHB blends (PLA:PHB) with different compositions (100:0, 90:20, 75:25, and 50:50) were prepared using extrusion temperature profiles ranging from 185 to 195 °C, as shown in Figure 1. The screw speed was 300 rpm, resulting into a throughput time of approximately 30–40 s. The blend combinations are shown in Table 1.

**Table 1:** Compositions of the Prepared PLA:PHB Blends and Nanocomposites

blend	PLA (wt %)	PHB (wt %)	GTA (wt %)	ChNC (wt %)
PLA	100			
90:10	90	10		
75:25	75	25		
50:50	50	50		
PHB		100		
nanocomposites				
(75:25):20	60	20	20	
(75:25):20:0.5	59.6	19.9	20	0.5
(75:25):20:1	59.2	19.8	20	1
(75:25):20:2	58.5	19.5	20	2

The nanocomposites were prepared via a liquid-assisted extrusion process using selected blend composition with 0.5, 1, and 2 wt % ChNCs aided by 20% GTA, as shown in Table 1. The suspension used for the liquid-assisted process was prepared by predispersing the concentrated ChNC gel (18 wt %) in ethanol at a ratio of 1:5 (water:ethanol) using magnetic stirring for 2 h because the used GTA plasticizer is not soluble in water.^33^ This was followed by the addition of the desired amount of the GTA and mixing with magnetic stirring. Finally, the suspension was ultrasonicated prior to extrusion. A schematic of the processing is shown in Figure 1, two atmospheric venting, and vacuum venting at the end of the process was used to ensure effective removal of the added liquid phase. The screw speed was 300 rpm resulting in a throughput time approximately 30–40 s.

The extruded materials were compression-molded to films using a Fontijne Grotnes LPC-300 hot press (Vlaardingen, Netherlands). Each material (approximately 3–4 g) was placed between two aluminum plates covered with Mylar (PET) films. Aluminum distances were used to obtain films with different thicknesses. Films with thicknesses in the 150–200 μm range were used for the studies of morphology, thermal, and mechanical properties and the films with thicknesses of approximately 50–70 μm were prepared for barrier properties studies. The materials were first preheated in press for 2 min at 190 °C at the contact pressure and then pressed at a constant pressure of 2 MPa for 1 min followed by cooling to 20 °C at a cooling rate of approximately 25 °C/min. All the films were stored for 1 week at room temperature before the characterizations to let the materials to rearrange and experience same history.

### Characterization

The spherulite morphology and crystallization rate of the produced materials were studied using a Nikon Eclipse LV100 Pol polarized optical microscope (BergmanLabora AB, Danderyd, Sweden) equipped with a Linkam THM600 (Tadworth, U.K.) hot stage. The film was first melted between two glass covers at 200 °C in the hot stage, followed by a cooling step to room temperature. Subsequently, the spherulite nucleation and growth were recorded using the microscope with an attached charge-coupled device (CCD) camera. POM was also used to study the crystallization rate of the optimal blend and its nanocomposites at an isothermal temperature of 90 °C for 30 min.

The morphology of the PLA, PHB, blends, and nanocomposites were studied using scanning electron microscopy (SEM) using a JEOL JSM-6460LV instrument (JEOL, Tokyo, Japan) at an acceleration voltage of 5 kV. The samples were cryo-fractured in liquid nitrogen and sputter-coated with a thin layer (∼10 nm) of platinum using an EM ACE200 Leica vacuum coater (Wetzlar, Germany) to avoid charging.

Thermal properties were examined using a TA Q500 thermogravimetric analyzer (TGA; TA Instruments, New Castle, DE, USA) in a nitrogen environment (flow rate of 60 mL/min) at a temperature range of 30–600 °C. The experiment was repeated at least 3 times for each material.

A Mettler Toledo differential scanning calorimeter DSC 822e, (Greifensee, Switzerland) was used to analyze the thermal properties of the compression molded films, including the crystallization temperature (*T*_c_), glass transition temperature (*T*_g_), cold crystallization temperature (Tcc), melting temperature (Tm), and the degree of crystallinity (*X*_c_). *X*_c_ was calculated using the Segal equation after baseline correction:^34^
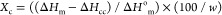
1where Δ*H*_m_ is the melting enthalpy, Δ*H*°_m_ = 93 J/g for PLA, and Δ*H*°_m_ = 146 J/g for PHB. Δ*H*_cc_ is the enthalpy of cold crystallization, Δ*H*°_m_ is the enthalpy of melting for fully crystalline PLA, and *w* is the weight fraction (6–10 mg) of the polymer. The material was first cooled to −40 °C and kept at this temperature for 3 min, and then heated to 230 °C at a heating rate of 10 °C/min. The sample was kept at 230 °C for 3 min, followed by cooling to −40 °C at a rate of 10 °C/min. This measurement was repeated twice.

A PANalytical Empyrean X-ray diffractometer (Almelo, The Netherlands) with Cu Kα radiation at a wavelength of 1.5405 Å (2θ range of 2–30°, steps of 2°/min) was used to determine the degree of crystallinity and the average crystal size using eqs 2 and 3, respectively. The crystal size and degree of crystallinity (*X*_c_) were evaluated by determining the intensities of the crystalline (*I*_c_) and amorphous (*I*_a_) contents in the sample using eq 2:
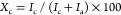
2

The average crystal size (*D*) was calculated using the Debye–Scherrer eq 3:^35^
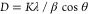
3where *K* is a constant close to unity, λ is the X-ray wavelength, θ is the Bragg angle, and β is the line broadening at half-maximum intensity (fwhm).

The mechanical properties of the samples were examined using a Shimadzu AG tensile tester (Kyoto, Japan). A rectangular press mold was used to cut the sample with a width and length of 5.5 mm and 50 mm, respectively. The samples were conditioned for 24 h at 25 °C at relative humidity (RH) of 50 prior to the testing. The gauge length, strain rate, and load cell were 20 mm, 2 mm/min, and 1 kN, respectively. The tensile strength and elongation at break are obtained from the testing software, and the toughness and tensile modulus were calculated from the stress–strain data.

Oxygen permeability (OP) was measured using an ADM2000 universal gas flow meter 2850 (Agilent Technologies, Wilmington, DE, USA) at 24 °C and 53% RH. The sample film disc (diameter = 25 mm) was clamped between two o-rings, and a constant oxygen gas flow at a pressure of 1 bar (75 cmHg) was applied to the film. The average exit flow rate (mL/min) from at least three samples was used for the calculation to determine the oxygen transmission rate (OTR) value using eqs 4 and 5:
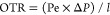
4
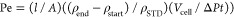
5where Pe is the permeability coefficient, *l* is the film thickness, *A* is the film contact area, ρ_end_ and ρ_start_ are the gas densities at the initial and final temperature and pressure conditions, respectively, ρ_STD_ is the density of the gas at standard temperature and pressure (*T* = 273.15 K and *P* = 1 bar), *V*_cell_ is the volume of the downstream chamber, Δ*P* is the pressure gradient between the chambers, and *t* is time. The film thickness varied between 40 and 63 μm.

The carbon dioxide transmission rate (CO_2_ TR) measurement followed the ASTM F2460-20 standard. The sample film disc (diameter = 50 mm) was clamped between the two o-rings of the gas cell with a total volume of ∼480 mL. One side of the cell (test side) was flushed with CO_2_ gas (purity, 99.99%), while the other side was flushed with N_2_ (purity, 99.99%), and the RH was <1%. The concentration of the CO_2_ diffused through the sample was measured relative to the N_2_ carrier gas by Fourier transform infrared spectroscopy (FT-IR, Gasmet DX4040) at room temperature (∼22 °C). For both gases, the gas flow was 50 mL/min as controlled by a tube flow meter for CO_2_ (model P11A2-BA0A) and a mass flow controller for N_2_ (Bronkhorst F-201DV-series). The measurement time ranged from ∼2 h up to several hours, depending on the time required for the stabilization of the CO_2_ concentration in the N_2_ carrier. The procedure was repeated for at least five samples, and the average value is presented.

## Results and Discussion

### Effect of PHB on the Crystallization

**Figure 2:**
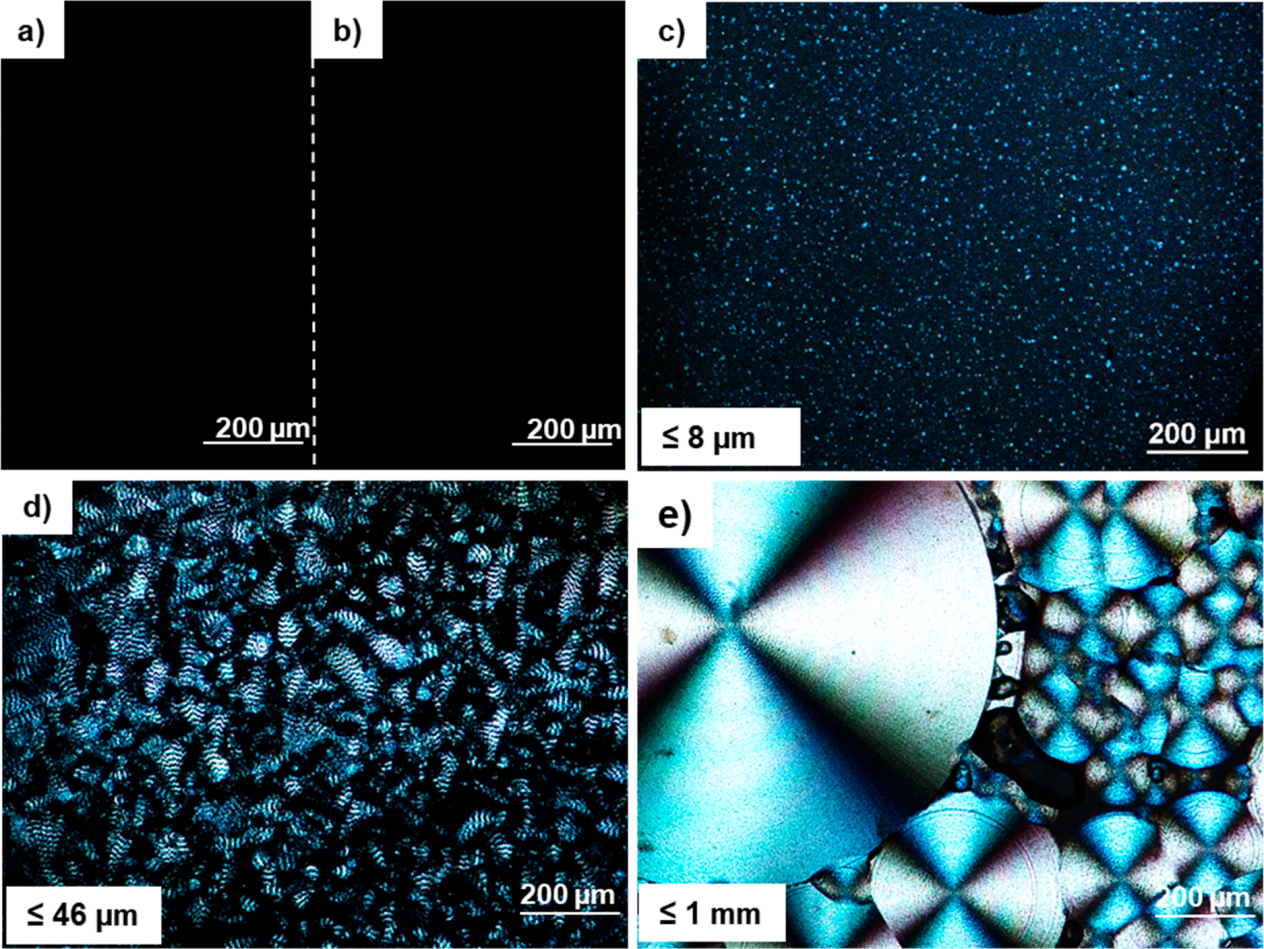
Polarized optical micrographs of melted films on the hot stage showing the crystallization of (a) pure PLA, (b) 90:10 with no visible crystallization, (c) 75:25 small crystallites well-dispersed in the PLA matrix, (d) 50:50 with large spherulites, and (e) pure PHB showing very large spherulite size compared to the blends.

The crystallization behavior and spherulitic morphology of the neat polymers and their blends were investigated using POM, with the results shown in Figure 2. The PLA shows no spherulites (Figure 2a) under the given conditions, confirming the amorphous structure of the PLA. In contrast, the PHB film exhibited a crystalline phase (Figure 2e) with large spherulites with a diameter of approximately 1 mm grown in helical strands radiating from the nucleation point. For the blends, mixing of PLA and PHB resulted in the formation of smaller spherulites, except for the 90:10 blend (Figure 2b), where the blend did not show any spherulites, possibly due to the low PHB content that is insufficient to produce spherulites. The 75:25 blend shown in Figure 2c demonstrates many very small spherulites (≤8 μm) that are well-dispersed in the matrix, whereas the 50:50 blend (Figure 2d) has a larger spherulite size (≤46 μm) and forms a continuous phase. This finding shows that the size of spherulites increased with the amount of PHB present in PLA. The formation of a large number of relatively small spherulites in the 75:25 blend may be ascribed to the number of nucleation sites of the spherulites that is high enough to obstruct the growth of large spherulites.^36^

SEM cross-sectional fracture surface images of the blends and neat polymers are presented in Figure S2 in the Supporting Information. The PLA exhibited a typical plane and brittle fracture surface, whereas the fracture surface of pure PHB is different, the fracture is brittle but there are no plane surfaces. The appearance of PLA and PHB fracture surfaces can be attributed to their amorphous and crystalline natures, respectively, as shown in the DSC study. Like PLA, the 90:10 blend exhibited an amorphous surface due to lower PHB content. In contrast, blending PLA with PHB results in a brittle fracture surface in the 75:25 and 50:50 blends. Very small, uniformly dispersed, and distributed white dots are observed in the 75:25 and 50:50 blends, possibly owing to the presence of PHB spherulites as revealed in the POM micrographs.

The thermal decomposition of neat PLA, PHB, and their blends are summarized in Table S1, and the corresponding thermograms are presented in Figure S3 in the Supporting Information. The neat PLA and PHB typically exhibited a single-step degradation process with a quite narrow decomposition temperature range, such as that previously reported by Arrieta et al.^18^ In contrast, the decomposition of the blends occurred in two well-separated steps. The first peak is related to the degradation of PHB, whereas the second stage is related to the degradation of PLA. These results confirmed that the presence of PHB decreased the onset degradation temperature (*T*_0_) of PLA in all of the blends. However, there was no indication of degradation at the temperatures below 200 °C, indicating that the PLA:PHB blends were thermally stable under the chosen extrusion process conditions without risking thermal degradation. The temperature corresponding to the maximum rate of weight loss (*T*_max_) of PLA was approximately 368 °C, while PHB is less thermally stable with a maximum degradation rate observed at approximately 289 °C. Hence, in the binary system, the addition of PHB decreases the thermal stability of the PLA:PHB blend.

The thermal properties obtained from the DSC curves such as the glass transition temperature (*T*_g_), melting temperature (*T*_m_), and degree of crystallinity (*X*_c_) are summarized in Table 2, in addition the exothermic peaks of neat PLA, PHB, and their blends for the first heating cycle and first cooling cycle are shown in Figure S4 (Supporting Information).

**Table 2:** Thermal Properties of Neat PLA, PHB, and PLA:PHB Blends Obtained from DSC

material	*T*_g_ (°C)	*T*_mPLA_ (°C)	*T*_mPHB_ (°C)	*T*_cc_ (°C)	*T*_c_ (°C)	*X*_c__PLA_ (%)	*X*_c__PHB_ (%)
PLA	55	142		103	77	2.7	
90:10	52	143	161	124	75	4.2	24
75:25	51	146	166	111	71	5.9	56
50:50	47	142	164		84	1.9	40
PHB			165		69		52

The *T*_g_ value was 55 °C for the neat PLA polymer, while the *T*_g_ peak was absent in the case of pure PHB; see the first heating scan (Figure S4a). Furthermore, the blending of PLA and PHB results in the shifting of the *T*_g_ peak toward a lower temperature compared to PLA. Similarly, the *T*_m_ values were 142 °C for PLA and 165 °C for PHB. The melting peak for PLA was contributed by the PLA crystals formed during the cold crystallization process, whereas the blends showed a double-melting behavior due to the melting of PLA (*T*_mPLA_) and PHB (*T*_mPHB_) at different temperatures. The presence of more than one melting peak indicates the lack of full miscibility between the polymers.^9,37^ For the 90:10 blend, only one sharp peak corresponding to the PLA melting component with a small shoulder peak for PHB was observed. For the 75:25 blend, sharp melting peaks at 146 and 166 °C were observed that correspond to the melting of the PLA and PHB components, respectively. In the case of the 50:50 blend, only one discernible peak related to the PHB melting peak was observed. During the cooling scan shown in Figure S4 b, only PHB exhibited a sharp crystallization peak (*T*_c_) at approximately 69 °C, indicating that pure PHB has higher overall crystallinity and crystallization rate than PLA.

The crystallinity of the used polymers and blends was evaluated using DSC and XRD analyses. Neat PLA exhibited amorphous behavior (2.7% crystallinity), whereas neat PHB showed crystalline behavior with 52% crystallinity. For the blends, the crystallinity of the PLA component (*X*_cPLA_) does not vary considerably, whereas the PHB crystallinity (*X*_cPHB_) varies significantly among the different compositions. Hence, the results clearly indicate that the spherulites formed in the POM studies were due to PHB. The 90:10 blends showed much lower crystallinity (*X*_cPHB_) compared to the other compositions, possibly due to a lower quantity of PHB that is insufficient for spherulite formation, as shown by the POM analysis. However, the addition of 25% or more PHB was sufficient to give rise to a higher crystallinity (*X*_cPHB_) of the resulting material. The 75:25 blend exhibited the highest degree of crystallinity for the PHB phase (56%), possibly due to the formation of many small well-dispersed PHB spherulites in the blend.

XRD spectra of the used polymers and blends are shown in Figure S5 in the Supporting Information. PLA exhibited a typical amorphous broad peak, whereas neat PHB showed two distinct diffraction peaks at 2θ of 13° and 17°, respectively, associated with the (020) and (110) planes of the orthorhombic unit cell and three weak peaks at 19.1°, 22.2°, and 25.5°, like the results previously reported for PHB.^38^ The peaks patterns of all PLA:PHB blends are quite similar to those of PHB, except for the 90:10 blend for which the diffractogram follows the typical PLA amorphous broad peak pattern, possibly due to the presence of a higher amount of PLA that likely limits the influence of the PHB. For the 75:25 and 50:50 blends, the intensity peaks of PHB are observed due to the higher crystallinity and the crystal growth rate of PHB, indicating that the addition of PHB significantly improves the crystallinity and the crystallization rate of the PLA:PHB blends.

The degree of crystallinity and average crystallite size were determined and are reported in Table S2 (Supporting Information). The values of the degree of crystallinity for the 75:25 and 50:50 blends were nearly the same and the crystallinity values were slightly lower than the values from the DSC analysis.

From the results for the first step of the two-step strategy in which different compositions of PLA and PHB were evaluated in terms of the degree of crystallinity, it was determined that the blend with 25 wt % PHB was the most suitable for the preparation of nanocomposites owing to its higher degree of crystallinity. The nanocomposites were prepared with the addition of different ChNC contents to study their effect on crystallization and biopolymer film properties.

### ChNCs as a Nucleation Agent in Nanocomposites

**Figure 3:**
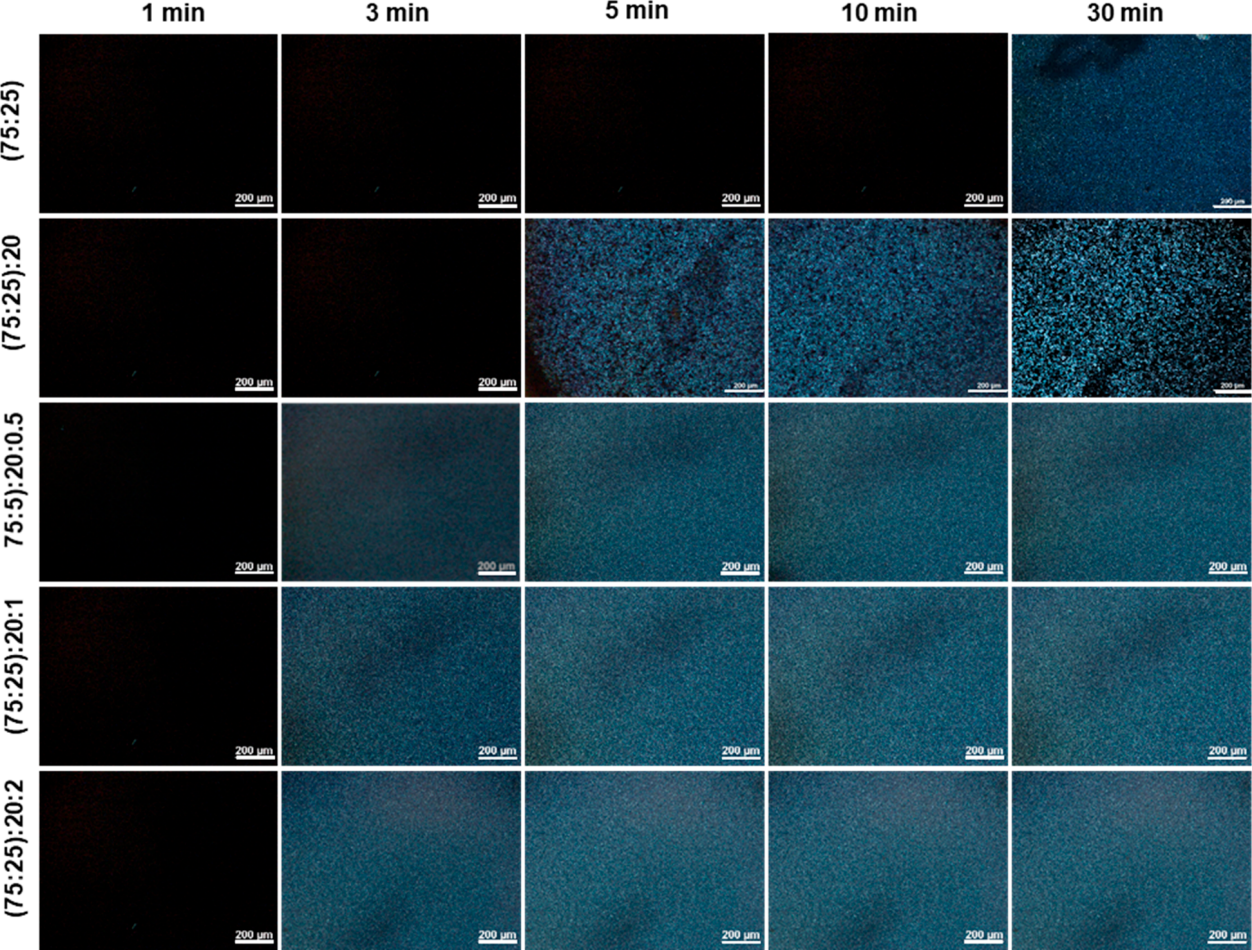
POM images of the PLA:PHB 75:25 blend, blend with GTA, and the nanocomposites with different ChNC contents (0.5, 1, and 2), obtained at an isothermal temperature of 90 °C for 1, 3, 5, 10, and 30 min (scale bar, 200 μm).

Figure 3 shows the micrographs of the PLA–PHB 75:25 blend and its nanocomposites at 90 °C obtained at different time intervals (1, 3, 5, 10, and 30 min). The isothermal temperature was selected where both polymers showed slow or no crystallization to study the effects of the interaction between the two polymers in the blends on crystallization. It is observed that spherulite formation occurs in the 75:25 blend in the time period of 10–30 min. The (75:25):20 samples, however, show spherulite formation in 3–5 min, indicating that the addition of GTA promotes spherulite formation, hence increasing the crystallization rate. According to previous studies, this phenomenon is mediated by the plasticizing effect that makes the polymer chains more flexible and facilitates the formation of spherulites. The addition of the ChNCs further increases the crystallization rate, and the spherulites are formed in 3 min.^24,39^

Notably, the nanocomposites containing ChNCs crystallize faster than the 75:25 and (75:25):20 samples where spherulites started to develop in the time period of 1–3 min. In fact, the nanocomposites containing 1 and 2 wt % ChNCs were nearly fully crystallized within 3 min. This is due to the significantly higher nucleating effect provided by ChNCs that favors the formation of PHB spherulites and results in a faster crystallization process. Similarly, in our previous study,^30^ it was reported that the addition of ChNCs improved the crystallization rate of PLA. Additionally, a distinct decrease in the average spherulite size is observed in the samples containing ChNCs due to the presence of many nucleation sites resulting in a large number of fine spherulites that do not allow the spherulites to grow to a large size. Furthermore, the spherulites in the nanocomposites are uniformly distributed, indicating no evidence of ChNCs agglomeration.

The influence of the addition of ChNCs and GTA on the thermal and crystallization properties of the PLA:PHB blends was investigated using isothermal TGA and DSC measurements, with the obtained results, and the derived parameters are summarized in Table 3 and presented in Figure S6 in the Supporting Information. The TGA in the isothermal mode was performed at 180 °C for 120 min to remove as much GTA as possible to prevent the interference between the GTA vaporization and PHB degradation peaks. The degradation peak from 100 °C to the isothermal region in the DTG curve (Figure S6 a) is linked to the vaporization of the GTA. The TGA curve revealed that the addition of GTA significantly decreased the thermal degradation temperature (*T*_0_) of the 75:25 blend. However, the addition of ChNCs counteracted the negative impact of GTA and led to the delay of the initialization of the thermal decomposition process in all cases. Additionally, the presence of ChNCs lead to a shift of the *T*_max_ of the 75:25 blends toward higher temperatures, and this behavior was particularly pronounced in the 75:25:20:1 blend, where the increase in *T*_max_ was approximately 57 °C (from 288 to 345 °C) for the PHB degradation peak and 61 °C (from 365 to 426 °C) for the PLA degradation peak. Moreover, the addition of ChNCs leads to a reduction in the vaporization peaks of the GTA in the isothermal temperature region due to its plasticizer antimigration capability, making it difficult to remove GTA at the isothermal temperature.^29^ These findings indicate that the ChNCs have a favorable impact on the nanocomposite performance, with the maximum improvement obtained in the 75:25:20:1 blend.

**Table 3:** Thermal Properties of PLA:PHB (75:25) Blends with GTA and ChNCs, Measured with TGA and DSC

	TGA			DSC					
		*T*_max_ (°C)							
material	*T*_0_ (°C)	step 1	step 2	*T*_g_ (°C)	*T*_mPLA_ (°C)	*T*_mPHB_ (°C)	*T*_cc_ (°C)	*X*_c__PLA_ (%)	*X*_c__PHB_ (%)
(75:25)	244 ± 2	288 ± 1	365 ± 2	51	143	166	111	5.9	56
(75:25):20	220 ± 2	287 ± 2	361 ± 3	30	127	158	88	3.7	52
(75:25):20:0.5	254 ± 1	335 ± 1	409 ± 1	38	136	155		5.7	63
(75:25):20:1	258 ± 0	345 ± 1	426 ± 0	46	134	173	103	1.5	86
(75:25):20:2	257 ± 1	335 ± 1	418 ± 1	46	134	168	93	1.4	78

The first DSC heating scans of the nanocomposites and their reference materials are shown in Figure S6b in the Supporting Information. Considering that GTA increases polymer molecular mobility, it was predicted that the plasticizing impact of GTA in the PLA:PHB (75:25):20 blend will result in a decrease in the glass transition temperature.^33^ The addition of ChNCs counteracted the effect of GTA, and a significant increase in the *T*_g_ value was observed for all nanocomposites, which was in good agreement with the previously reported results for plasticized PLA materials.^14,29^*T*_cc_ and *T*_m_ show a tendency similar to those of the *T*_g_ values; first, the addition of GTA decreased the *T*_cc_ and *T*_m_ values of the (75:25):20 samples, but these values were then increased significantly with the addition of ChNCs. The addition of the GTA plasticizer considerably lowered the *T*_m_ values of PLA and PHB, confirming its effectiveness as a plasticizer for both PLA^14,33^ and PHB.^40^ Here, it should be highlighted that the nanocomposite with 1 wt % ChNCs showed significant improvements, with the *T*_g_ increased from 30 to 46 °C, and the *T*_cc_ increased from 88 to 103 °C.

The results for the degree of crystallinity of both PLA and PHB components in the prepared materials are summarized in Table 3. It is observed that the crystallinity of the PLA content did not show any improvement with the addition of ChNCs while the degree of crystallinity for PHB increased significantly, indicating that the PHB crystallization rate is higher and utilizes all available ChNCs to form the PHB spherulites around the ChNCs. The synergistic effect on the crystallization of the polymer blend due to the plasticizer and the potential nucleating agent was previously observed by Arrieta et al.^10^ in a PLA:PHB blend reinforced with cellulose nanocrystals and plasticized with acetyl tributyl citrate (ATBC). For the (75:25):20:1 blend, the addition of 1 wt % ChNCs led to the highest degree of crystallinity among all nanocomposites because the improved dispersion of the ChNCs in (PLA:PHB):GTA provides a large number of nucleation sites for the formation of PHB spherulites. The higher crystallinity achieved by the (75:25):20:1 sample is due to the presence of the optimal quantity of GTA on the surface of the nanocrystal that leads to better dispersion and therefore an enhanced nucleation effect. To summarize, the addition of ChNCs improved the thermal characteristics and the degree of crystallinity, with the (75:25):20:1 sample, in particular, exhibiting clearly superior performance.

**Figure 4:**
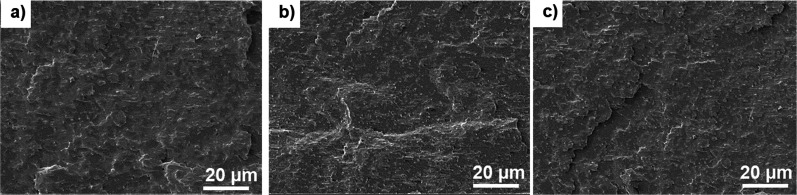
Fracture surface images of the PLA:PHB:GTA:ChNC nanocomposites with ChNC contents of (a) 0.5 wt %, (b) 1, and (c) 2 wt %, showing uniform dispersion of the ChNCs and no evident large agglomerates.

To investigate the dispersion of ChNCs in the nanocomposites, SEM was performed to examine the microstructure of the prepared nanocomposite cryo-fracture surfaces, with the results shown in Figure 4. All nanocomposites exhibit a highly brittle surface, most likely due to their high crystallinity, and no evident ChNC agglomerates are observed in any of the nanocomposites’ fracture surfaces, suggesting that the liquid-assisted extrusion approach associated with the GTA plasticizer resulted in good ChNC dispersion in all three nanocomposites. However, according to Bondeson and Oksman,^41^ SEM investigation of the dispersion of a low concentration of nanomaterials in the matrix is difficult due to the low contrast between the PLA and bio-nanomaterial.

**Figure 5:**
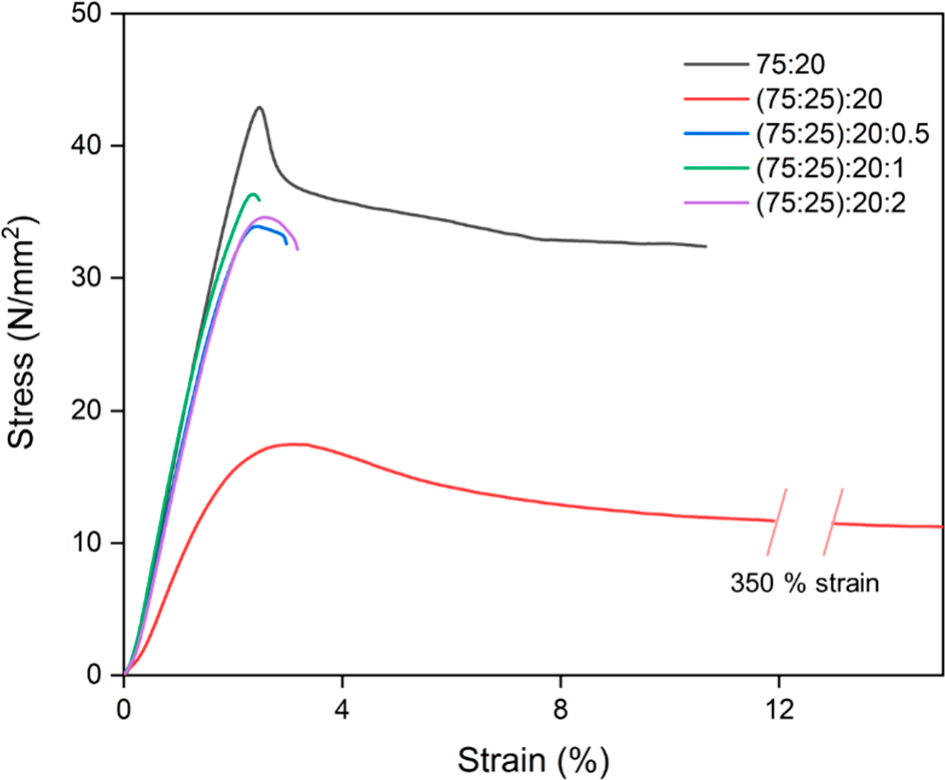
Mechanical properties of the 75:25 blend, the 75:25 blend with GTA, and the corresponding nanocomposites.

Since the crystalline morphology of the material is linked to its mechanical performance, the mechanical properties of the materials were investigated. The stress–strain curves are depicted in Figure 5, and the results are summarized in Table S3 in the Supporting Information.

The 75:25 blend outperformed the neat PLA and PHB in terms of mechanical properties, indicating that the finely dispersed PHB crystals act as a filler for the PLA matrix. Considering the mechanical properties of the 75:25 blend as reference material, it is observed that GTA addition substantially increased the elongation at break by a factor of 25, accompanied by a reduction in Young’s modulus and tensile strength. These results are attributed to the strong plasticizing effect that was previously observed in the PLA-GTA,^33^ PLA-TEC^42^ and PHB-GTA^40^ systems.

The addition of ChNCs counteracted the negative impact of GTA by increasing Young’s modulus and tensile strength in each of the three nanocomposites owing to the positive effect of the ChNCs reinforcement. In particular, the incorporation of 1 wt % ChNCs significantly improved Young’s modulus (1.91 GPa), obtaining a value close to that of the reference material. This finding can be explained by the increased crystallinity (*X*_cPHB_) and intrinsic properties of better-dispersed ChNCs in the (75:25):20:1 material. Also, all nanocomposites demonstrated an increase in tensile strength in comparison to the (75:25):20 blend. However, the higher crystallinity of the PHB component resulted in a significant reduction in the elongation at break in all of the nanocomposites, making the material hard and brittle. As a result, all of the nanocomposites exhibited lower toughness values than that of the (75:25):20 blend. But, this effect was most pronounced in the nanocomposite with 1 wt % ChNCs ((75:25):20:1) due to the highest degree of crystallinity, having a slightly lower toughness value (0.4 MJ/m^3^) among all of the nanocomposites.

A material used for food packaging applications must protect the food from the outside environment to limit the oxidation processes. Therefore, the OTR and the carbon dioxide transmission rate (CO_2_TR) of the films were determined with the results summarized in Table 4. PLA exhibited moderate oxygen barrier properties with an OTR value of 1853 cm^3^ m^–2^ day^–1^, whereas PHB is known for its higher barrier performance, and it exhibited an OTR value of approximately 626 cm^3^ m^–2^ day^–1^. Gas transport depends on the free volume of the polymeric material with more flexible chains, resulting in greater free volume, which in turn results in increased mass transport of gas molecules.^43^ Hence, the addition of the GTA plasticizer is expected to enhance the free volume in the polymer material and is likely to negatively affect the barrier performance of the 75:25 blend. The free volume is strongly affected by the degree of crystallinity of the film and impermeable nanomaterial, because of which, the gas molecules follow a tortuous path, and as a result, reduce the rate of the permeation process. Accordingly, the addition of ChNCs shows a 2-fold effect on the OP of the nanocomposites that are generated simultaneously by their inherent barrier properties and by the inducement of a higher degree of crystallinity. Hence, the highest crystallinity and better-dispersed ChNCs in the nanocomposite with 1 wt % ChNCs (75:25):20:1 exhibited the lowest OTR value (385 cm^3^ m^–2^ day^–1^) among all of the prepared nanocomposites; this value is approximately 4.8 times lower than the OTR value of PLA. Similarly, the addition of ChNCs decreased the CO_2_TR value; for instance, the CO_2_TR value for the nanocomposites with 0.5 wt % ChNCs is 520 cm^3^ m^–2^ day^–1^, which is 26% lower than that of the reference PLA:PHB blend. Additionally, the nanocomposites with 1 and 2 wt % ChNCs showed 40 and 52% lower values, respectively, compared to the (75:25):20 material, indicating that the addition of ChNCs improves the CO_2_TR performance.

**Table 4:** Oxygen and Carbon Dioxide Barrier Properties of PLA, PHB Biopolymers, Their 75:25 Blend, and Blend with GTA and Nanocomposites

material	OTR (cm^3^ m^–2^ day^–1^)	CO_2_ TR (cm^3^ m^–2^ day^–1^)
PLA	1853	
PHB	626	
(75:25):20	810	706 ± 54
(75:25):20.0.5	586	520 ± 14
(75:25):20:1	385	420 ± 4
(75:25):20:2	412	338 ± 21

## Conclusions

Crystallization and barrier properties of PLA were substantially improved via a two-step approach, involving melt blending of PLA with PHB and the use of ChNCs as the nucleation agent. In the first step, a range of PHB concentrations (10, 25, and 50 wt %) was blended with PLA to determine the optimal blend ratio that resulted in the highest crystallinity. Analysis of the blend morphology and crystallinity revealed that the 75:25 PLA:PHB blend showed a large number of small PHB spherulites in the amorphous PLA matrix and exhibited the highest crystallinity. Therefore, this composition was chosen as the matrix for chitin nanocomposites.

The liquid-assisted extrusion process was used to fabricate nanocomposites with varying concentrations (0.5, 1, and 2 wt %) of ChNCs as well as 20 wt % GTA as a processing and dispersing aid. The effects of GTA and ChNCs on the crystallization, morphological, thermal, mechanical, and barrier properties of the 75:25 blend were studied.

A polarized optical microscopy study of the crystallization of chitin nanocomposites showed that the ChNCs acted as a nucleation agent for the PHB phase and resulted in well-dispersed PHB spherulites in the PLA phase and a significant increase in the crystallization rate and degree of crystallinity. The highest effect was observed for the nanocomposites with 1 wt % ChNCs.

GTA had a detrimental influence on the thermal properties, but the addition of 1 wt % ChNCs was able to counterbalance this effect. The good dispersion of the ChNCs was confirmed by microscopy and no agglomerates were visible, indicating that the manufacturing process was successful.

The mechanical properties of the blends showed that the blending of 25 wt % PHB improved the mechanical properties of the 75:25 blend. However, the introduction of the GTA dispersion aid in the 75.25 blend significantly decreased the stiffness and strength of the material due to the plasticizing effect, whereas ChNCs had a positive effect on all nanocomposites; mechanical properties of all nanocomposites improved.

Substantial improvement in the crystallinity and homogeneous spherulite formation was reflected in the excellent barrier characteristics of the nanocomposites. The nanocomposite with 1 wt % ChNCs showed a reduction in the OTR value by 4.8 times compared to the neat PLA and by 1.6 times compared to neat PHB. Furthermore, the addition of ChNCs improved carbon dioxide barrier performance. These results suggest that the PLA:PHB with the 75:25 composition reinforced with ChNCs is a promising biopolymer for food packaging.

## Supporting Information Available

(Figure S1) AFM phase and height images and width and length ranges plots; (Figure S2) fractured surfaces micrographs; (Figure S3) TGAs; (Figure S4) DSC thermoscans; (Figure S5) X-ray diffraction patterns; (Figure S6) TGA and DTG thermograms and DSC scan; (Table S1) TGA analysis; (Table S2) crystal analysis results; (Table S3) mechanical properties (PDF)
