# Supplementary material for: Biopolymer Blends of Poly(lactic acid) and Poly(hydroxybutyrate) and Their Functionalization with Glycerol Triacetate and Chitin Nanocrystals for Food Packaging Applications
Source: ACS Appl Polym Mater. 2022 Aug 16;4(9):6592–601. doi: 10.1021/acsapm.2c00967 (PMC9469702; doi:10.1021/acsapm.2c00967)
Supplement: Supplementary file 1 — ap2c00967_si_001.pdf [file ap2c00967_si_001.pdf]

Supporting Information

Biopolymer blends of poly(lactic acid) and poly(hydroxybutyrate) and their functionalization with glycerol triacetate and chitin nanocrystals for food packaging applications

Mitul Kumar Patel,[†] *Freja Hansson,*[†] *Olli Pitkänen,* [‡] *Shiyu Geng* [†] *and Kristiina Oksman* [†,§, ‖*]

[†] Division of Materials Science, Department of Engineering Sciences and Mathematics, Luleå University of Technology, SE-97 187 Luleå, Sweden

[‡] Microelectronics Research Unit, Faculty of Information Technology and Electrical Engineering, University of Oulu, Oulu, Finland

[§] Mechanical & Industrial Engineering (MIE), University of Toronto, Toronto, ON M5S 3G8, Canada

‖ Wallenberg Wood Science Center (WWSC); Luleå University of Technology SE 97187 Luleå, Sweden

*Corresponding author: Kristiina Oksman (Email: kristiina.oksman@ltu.se; Tel: +46-(0)920-493371), Luleå University of Technology, SE-97 187 Luleå, Sweden

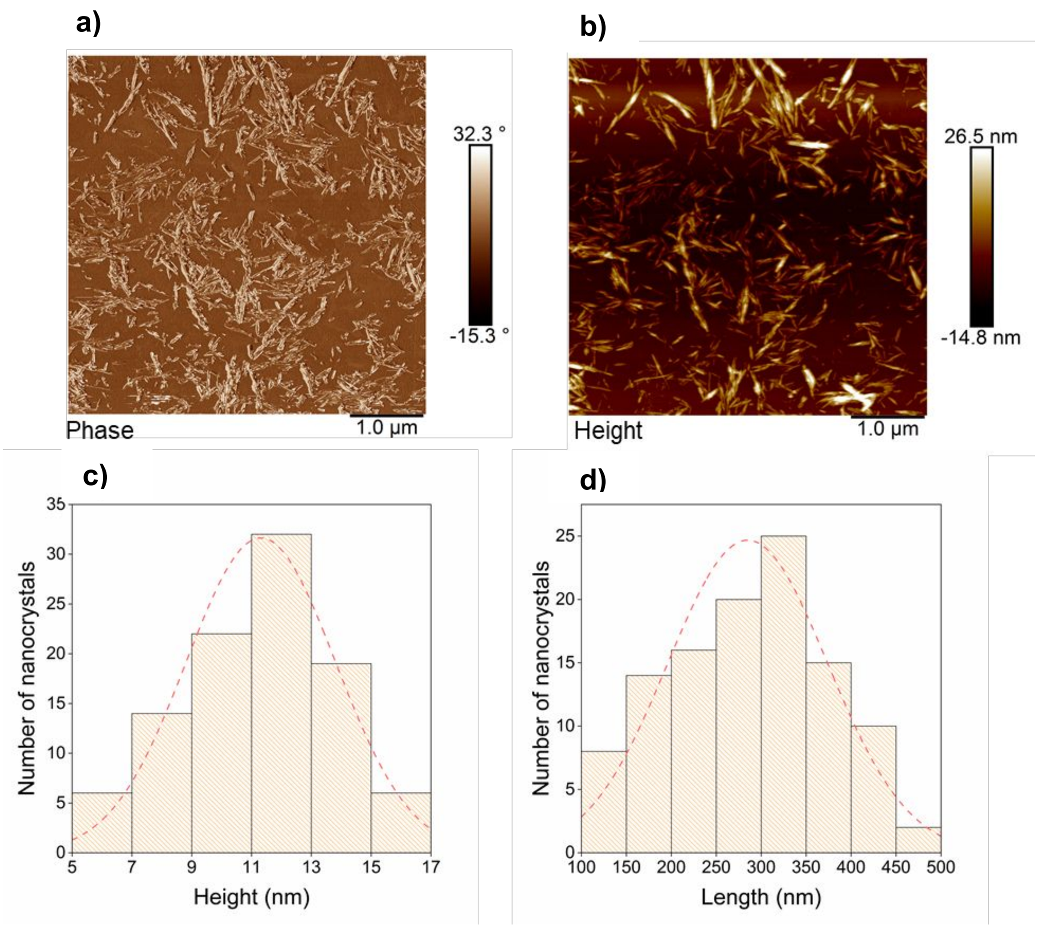

Figure S1. a) AFM phase image, b) height image of the produced ChNCs, c) width range between 5-16 nm (average 12 nm), and d) length range between 120-480 nm (average 286 nm).

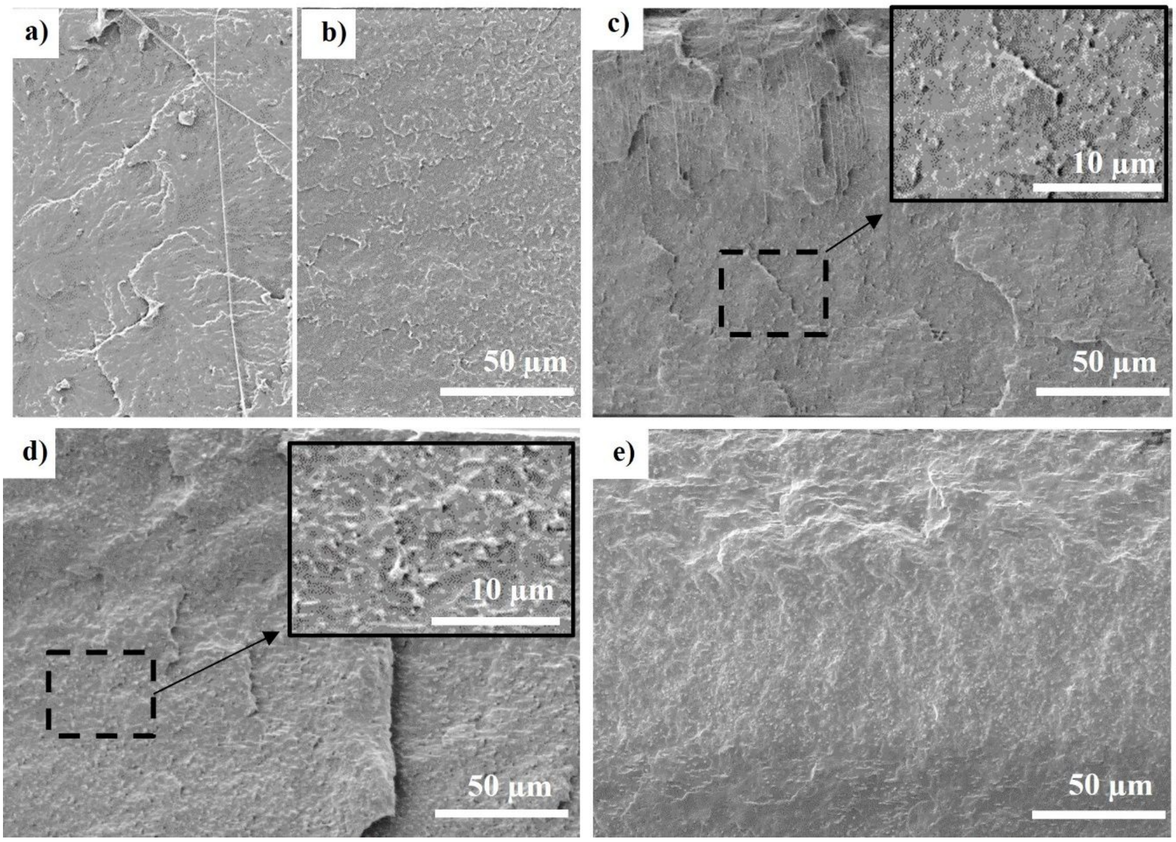

Figure S2. Micrographs of the fracture surfaces of the PLA, PHB and their blends. a) neat PLA b) 90:10, c) 75:25, d) 50:50 e) neat PHB.

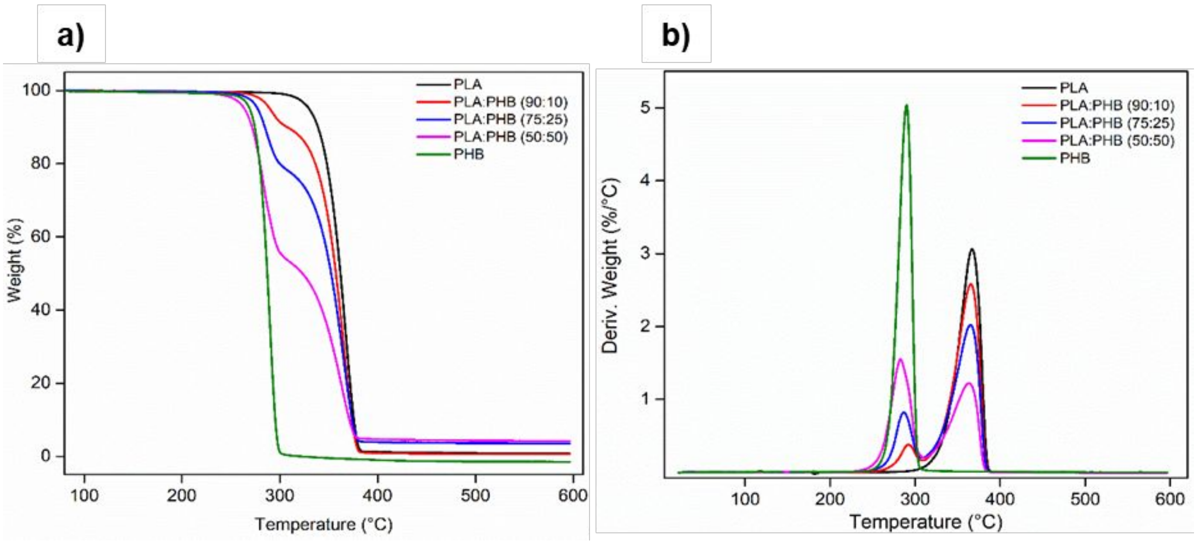

Figure S3. Thermal analysis of neat PLA, PHB and PLA:PHB blends. a) TGA and b) DTG curves.

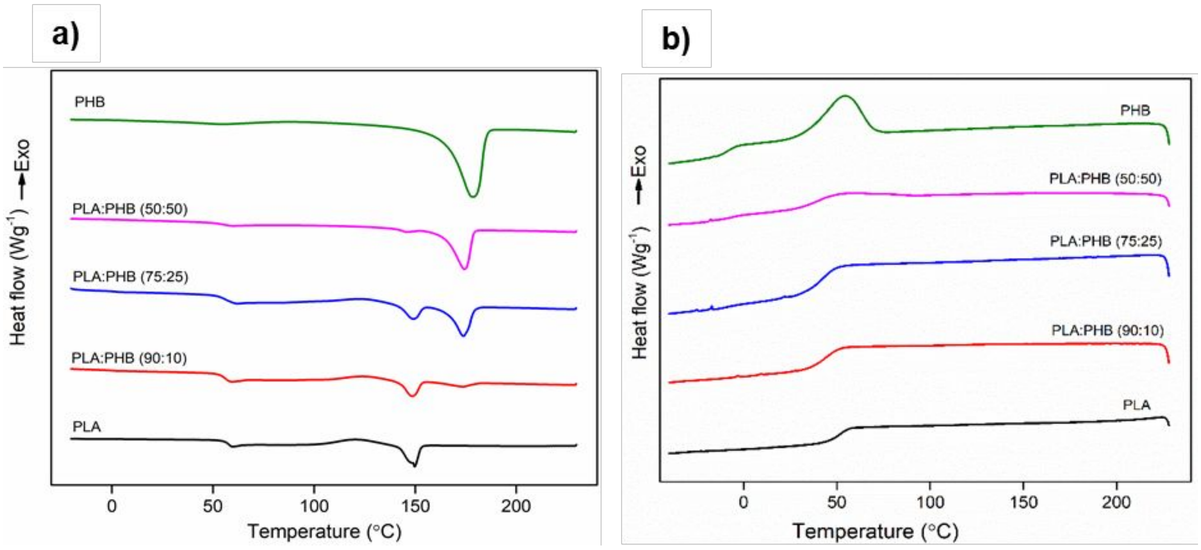

Figure S4. DSC thermograms; a) 1st heating scans b) 1st cooling scans of neat PLA, PHB and PLA:PHB blends.

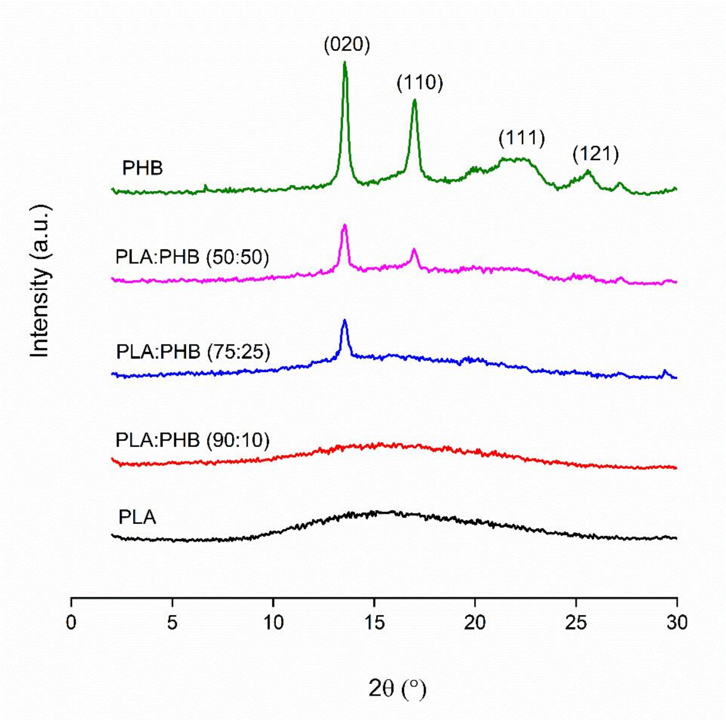

Figure S5. X-ray diffraction patterns of neat PLA, PHB, and PLA:PHB blends.

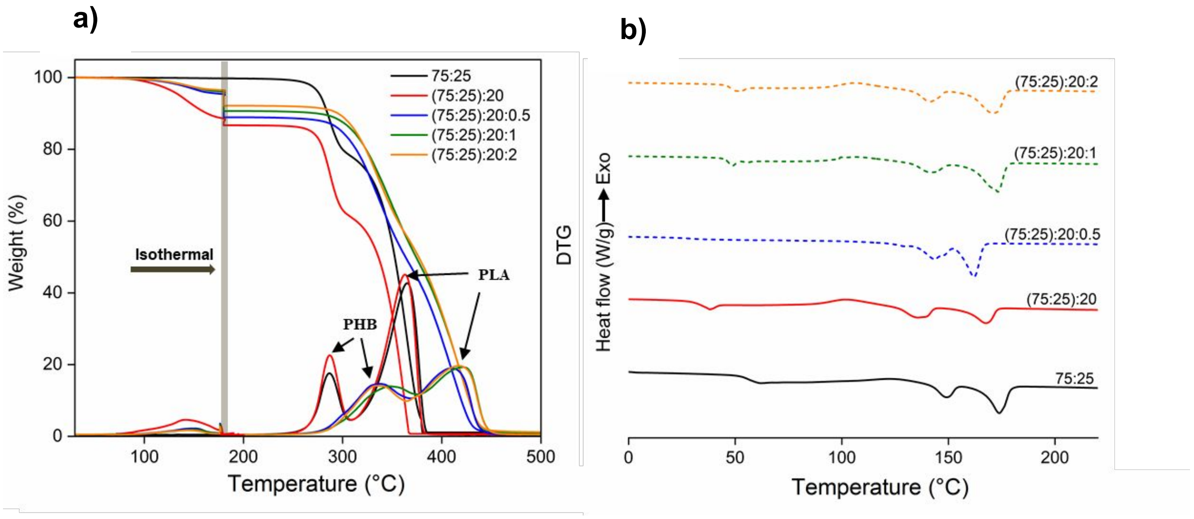

Figure S6. a) TGA and DTG thermograms of PLA:PHB 75:25 blend, and the blends with GTA and ChNCs, b) DSC thermograms (first heating scan) of 75:25 blend and its nanocomposites.

Table S1. TGA of neat PLA, PHB and PLA:PHB blends.

Materials	T_0 (˚C)	T_{max} (˚C)	
		Step 1	Step 2
PLA	296 ± 0	-	368 ± 1
90:10	250 ± 1	290 ± 0	373 ± 1
75:25	244 ± 2	288 ± 1	365 ± 1
50:50	224 ± 1	282 ± 2	363 ± 1
PHB	236 ± 0	289 ± 0	-

Table S2. Degree of crystallinity and crystallize size from XRD for PHB and PLA:PHB blends.

**Materials**	**Crystallinity (%)**	**Crystallite size (nm)**
75:25	45	16
50:50	45	21
PHB	52	24

Table S3. Mechanical properties of neat PLA, PHB, PLA:PHB 75:25 blend, 75:25 blend with GTA and its nanocomposites.

**Materials**	**Young's modulus (GPa)**	**Tensile strength (MPa)**	**Elongation at break (%)**	**Work of fracture (MJ/m^3)**
PLA	1.9 ± 0.0	55 ± 2	3.7 ± 0.2	1.6 ± 0.5
PHB	1.6 ±0.2	22 ± 2	2.1 ± 0.8	0.3 ± 0.0
75:25	2.0 ±0.1[a]	42 ± 2[a]	12.5 ± 4.5[a]	1.7 ± 0.3[a]
(75:25): 20	0.9 ±0.0[b]	21 ± 2[b]	316 ± 71[A]	47 ± 14[A]

(75:25): 20:0.5	1.7 ± 0.1^{c}	35 ± 3^{c}	2.8 ± 0.3^{b}	0.5 ± 0.1^{b}
(75:25): 20:1	1.9 ± 0.0^{a}	36 ± 1^{c}	2.5 ± 0.2^{b}	0.4 ± 0.0^{b}
(75:25): 20:2	1.8 ± 0.0$^{a, c}$	36 ± 1^{c}	3.4 ± 0.4^{b}	0.7 ± 0.1^{b}

*Note: The same superscript letters within the same column and material are not significantly different at the 5% significance level based on ANOVA and Tukey's HSD multiple comparison test.
